# Is soft tissue repair a right choice to avoid early dislocation after THA in posterior approach?

**DOI:** 10.1186/s12893-017-0212-3

**Published:** 2017-05-19

**Authors:** Yiqin Zhou, Shiqi Cao, Lintao Li, Manoj Narava, Qiwei Fu, Qirong Qian

**Affiliations:** 1 Joint Surgery and Sports Medicine Department, Shanghai Changzheng Hospital, Second Military Medical University, 415 Fengyang Road, Shanghai, 200003 China; 2 Department of Orthopedics, Jinling Hospital, Nanjing University School of Medicine, Nanjing, China; 3 University of Pittsburgh, Pittsburgh, USA

**Keywords:** Primary total hip arthroplasty, Soft tissue repair, Early dislocation

## Abstract

**Background:**

Dislocation is the second most common complication after total hip arthroplasty (THA). The effectiveness of soft tissue repair to reduce dislocation rate is still debated and thus a meta-analysis was conducted.

**Methods:**

A systematic search in PubMed, Embase, and Cochrane databases was conducted for this meta-analysis. Inclusion criteria: clinical comparative trials on the use of soft tissue repair including rotators and capsule repair in primary THA. The main data outcome were the incidences of early hip dislocation after primary THA. HSS score, incidence of other complications was also included in the outcomes.

**Results:**

A total of 4816 cases were included for the analysis from ten studies (3 RCTs/7 Retrospective trials). Overall, the soft tissue repair group showed a significant lower early dislocation rate and higher HSS score compared to the no repair group; but no significant difference was observed between the two groups in regards to the early dislocation rate in RCT studies only. The capsule repair group showed a significant lower early dislocation rate than no capsule repair group while no significant difference was observed between the rotators repair group and no rotators repair group. In all included studies, 4 greater trochanter fractures, 2 sciatic nerve palsies and 1 infection were reported in soft tissue repair group while no cases were observed in the no repair group.

**Conclusions:**

The efficacy of soft tissue repair is positive but still not conclusive to reduce the early dislocation rate after primary THA while soft tissue repair may bring more other complications. Capsule repair seems more effective than rotators repair only.

## Background

Dislocation is one of the most common complications after total hip arthroplasty (THA), an incidence second to septic loosening [1]. Most dislocations occurred 6 months after surgery and were referred to as early dislocations. Woo et al. from Mayo Clinic Center reported the dislocation rate in a 10500 research cases was 3.2% [2]. Demos reported dislocation rate after primary THA was 3.2 –6.5% while the incidence increased to 7.4–11.4% after revision [3]. The dislocation could cause a major physical and mental burden to patients, so it is important for surgeons to figure out better ways to decrease the incidence of dislocation.

In recent years, a few studies reported that surgeons tend to suture more soft tissue including the capsule and rotators (piriformis, superior gemellus, obturator internus, inferior gemellus) to decrease the dislocation rate when the posterior approach was taken in the primary THA [4–13]. However, the results were not consistent with each other and the effectiveness of soft tissue repair is still debated. Therefore, we did a meta-analysis to examine the efficacy of soft tissue repair in primary THA.

## Methods

This article was reported in accordance with the Preferred Reporting Items for Systematic Reviews and Meta-Analyses (PRISMA) guidelines.

### Identification and study selection

We conducted a literature search using PubMed (Medline), Embase and Cochrane Central Register of Controlled Trials to identify all articles published between 1966 and 2016 that evaluated the early dislocation rate of the posterior approach of primary THA with and without soft tissue repair (including joint capsule and external rotators). The key terms used were “Arthroplasty/Replacement”, “Hip”, “soft tissue repair/capsule repair/rotators repair”, and “dislocation”. A literature search was conducted for each author of the studies to find other relevant studies. Each study and previous review were examined manually to find further studies on similar topics. As we did not include unpublished research, publication bias could not be avoided.

In consideration of the selection bias, criteria for inclusion were as follows: English published papers and Chinese published papers whose English abstract could be searched in the database described above (Table 1). There was no history of hip infection or hip surgery associated with any of these cases. Although randomized controlled trials may be preferable in meta-analysis, number of such suitable RCT studies were limited, so reports were also considered suitable if they were comparative trials between THA with and without repair posterior soft tissues; all THA were of posterior approach of primary THA.

**Table 1:** Inclusion and exclusion criteria

Inclusion criteria	Exclusion criteria
Published papers in PubMed (Medline), Embase or Cochrane Central Register of Controlled Trials	Papers in languages other than English or Chinese whose English abstract could be searched in the database
Papers in English or Chinese whose English abstract could be searched in the database	THA in other approaches
Randomized controlled trials or clinical comparative trials	Case series or case report
Comparing soft tissue repair including rotators or capsules repair with no soft tissue repair in THA	
THA in posterior approach	

### Outcome assessment

Majority of the results were the incidences of early hip dislocation after primary THA. HSS score, operation time, incidence of other complications, and incidence of revision were also included in the outcomes. Titles and abstracts were judged first; then the full text was obtained and examined if the outcome was potentially eligible.

### Data extraction

After all suitable studies were chosen, data extraction was independently done by three authors subsequently. We gathered several kinds of component from these suitable studies including type of study, surgery methods, early dislocation rate, operation time, HSS score, age, follow-up, abduction angel, anteversion angle and complications.

### Data analysis and quality assessments

All analysis was performed using Review manager 5.3 and Stata 12.0. Relative risk (RR) was used for dichotomous outcomes, and the mean differences (MD) was used for continuous outcomes. The results were shown as forest plots. We used 95% confidence intervals (CI) for each study. I^2^ was used to estimate total variation across studies. A Chi text (*X* ^2^) significance level of less than 0.10 was interpreted as evidence of heterogeneity. While a random effect model was chosen when there was statistical evidence of heterogeneity, a fixed effect model was applied when there was no statistical evidence of heterogeneity. Additionally, meta regression was performed to assess the effect of confounding factors in the occurrence of dislocation. The regression was considered statistically significant if the *P* <0.05 level at the 95% CI did not intersect the midline.

The quality of the eligible studies was estimated according to the items recommended in Cochrane Collaboration (Revman 5.3; http://handbook.cochrane.org/), including selection bias, performance bias, attrition bias, detection bias, reporting bias, and other sources of bias. Two authors (ZY and CS) independently made the assessment of the quality of all the studies. Any disagreements were resolved by discussion, and finally judged by QQ.

## Results

**Fig. 1:**
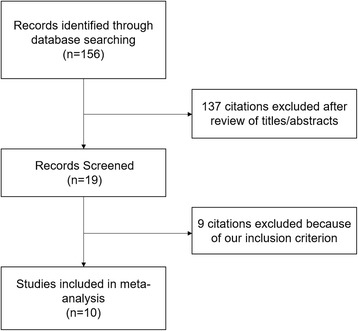
Flow diagram of study selection

A total of 156 potential studies were identified using the databases (71 from PubMed, 75 from EmBase, 10 from CNKI, and none from the Cochrane databases). Of these, 81 were excluded on the basis of the titles alone, 56 reports were excluded after review of the abstracts. 9 studies were excluded after detailed review of the full text. Ten studies were included in this meta-analysis (Fig. 1).

Table 2 summarizes the characteristics of the ten studies, which were published between 1998 and 2012. There were three studies of RCTs. The other seven were all retrospective comparative trial. A total of 4816 cases were finally included for the analysis. We examined the effect of soft tissue repair on the early dislocation rate, HSS score and operation time. We also examined the subgroup results to determine the effect of capsule repair only or rotators repair only had on the early dislocation rate, and the effect of soft tissue repair on the early dislocation rate in RCTs only.

**Table 2:** Characteristics of included studies

Article	Region	Language	Type	Group	Rotators Repair	Capsule Repair	Case	E-D	O-T	Post-HSS	Age	F-U (month)	ABA	ANA	O-C
Pellicci 1998 [4]	North American (USA)	English	Retro, single	I	yes	yes	519	1	N/A	N/A	N/A	6–12	N/A	N/A	
				II	yes	no	395	16				12			
				III	no	no	160	10				6			
Chiu 2000 [5]	Asia (Taiwan)	English	RCT, single	I	yes	yes	96	0	N/A	93 (86–96)	51 (25–84)	38(12–60)	40°	15° + 10°	
				II	no	no	84	2		91 (86–95)	54 (29–90)	38(12–60)	40°	15° + 10°	
White 2001 [8]	North American (USA)	English	Retro, single	I	yes	yes	434	3	N/A	N/A	N/A	>6	45.2°	22.9°	4 greater trochanter fracture
				II	no	no	1081	52				>6	44.5°	22.7°	
Goldstein 2001 [6]	North American (USA)	English	Retro, single	I	yes	yes	500	3	N/A	N/A	N/A	12	N/A	N/A	
				II	yes	no	500	14				12			
Suh 2004 [10]	Asia (Korea)	English	Retro, single	I	yes	yes	96	1	120.3 ± 7.8	95.2 + −3.3	53.3 ± 10.8	12	45° ± 2°	23° ± 5°	
				II	no	no	250	16	118.4 ± 8.3	93.9 + −5.5	53.5 ± 10.4	12	44° ± 3°	22° ± 5°	
Tarasevicius 2006 [11]	Europe (lithuania)	English	RCT, single	I	yes	yes	14	0	N/A	N/A	70 ± 5.7	over 12	N/A	N/A	
				II	no	no	13	1			70.6 ± 8.5	over 12			
Kim 2008 [9]	Asia (Korea)	English	Retro, single	I	Yes	Yes	282	11	90.1 ± 11.6	94.9 ± 3.4	47.6 ± 13.8	12	42.2° ± 3.6°	15.3° ± 5.5°	1 infection
				II	no	no	168	9	81.2 ± 14.1	94.2 ± 3.2	49.2 ± 15.1	12	42.5° ± 3.7	15° ± 4.9	
Tsai 2008 [7]	Asia (Taiwan)	English	Retro, single	I	yes	yes	62	0	N/A	N/A	63 ± 13.31	14.8	37.4° (33°–41°)	11.1° (5°–14°)	
				II	yes	no	142	9			58 ± 14.06	51.6	38.16° (30°–43°)	9.86° (4°–15°)	
Tarasevicius 2010 [12]	Europe (lithuania)	English	RCT, single	I	yes	yes	135	3	N/A	N/A	69 ± 8	>12	30–55°	N/A	2 sciatic nerve palsies
				II	no	no	141	7			68 ± 9	>12	30–55°		
Shen 2012 [13]	Asia (China)	English absract/Chinese	Retro, single	I	yes	yes	38	0	79.97 ± 5.09	95.66 ± 4.51	67.6 (58–79)	>12	40 ± 10	15 ± 10	
				II	no	yes	39	0	81.44 ± 5.89	95.49 ± 2.81	70.1 (59–81)	>12			
				III	yes	no	41	4	80.24 ± 5.83	94.98 ± 3.09	69.3 (58–82)	>12			
				IV	no	no	41	4	81.24 ± 3.91	95.37 ± 3.23	68.7 (57–82)	>12			

*E-D* early dislocation, *O-T* operation time, *F-U* Follow-up, *ABA* Abduction angle, *ANA* Anteversion angle, *O-C* other complication (except dislocation), *NA* not applicable/mentioned

**Fig. 2:**
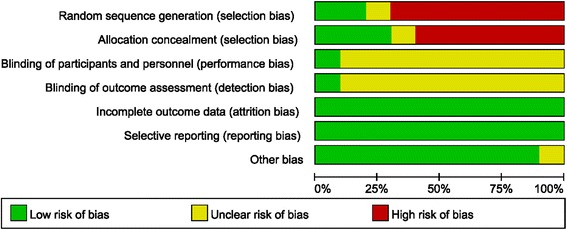
Summary graph of author judgments for each risk of bias criteria

**Fig. 3:**
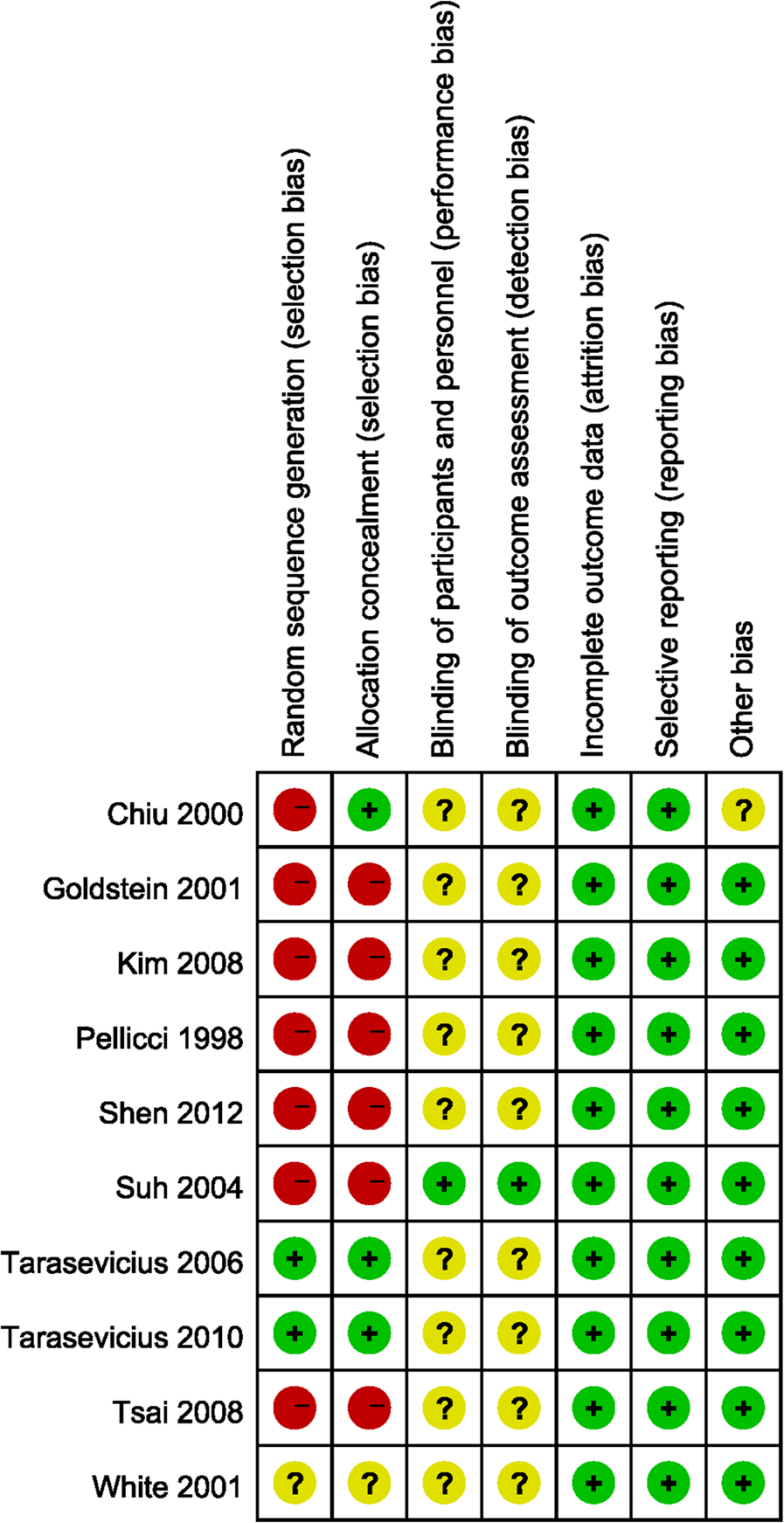
Risk of bias assessment based on author judgment for individual studies

Characteristics and quality of all included studies are presented in Figs. 2 and 3. The methods of random allocation were described clearly in only 3 trials, including 1 quasi-randomized controlled trial. Only 1 study was described as being inconclusive to patients and doctors. No data was incomplete and selective report didn’t exist in all included studies. Inter-rater reliability for the risk of bias assessment was calculated, yielding a κ-statistic of 0.69 (*p* < 0.01) and Kendall W of 0.977 (*p* = 0.04), indicating good agreement between raters.

### Result 1: Comparison of early dislocation rate between soft tissue repair group and no repair group

**Fig. 4:**
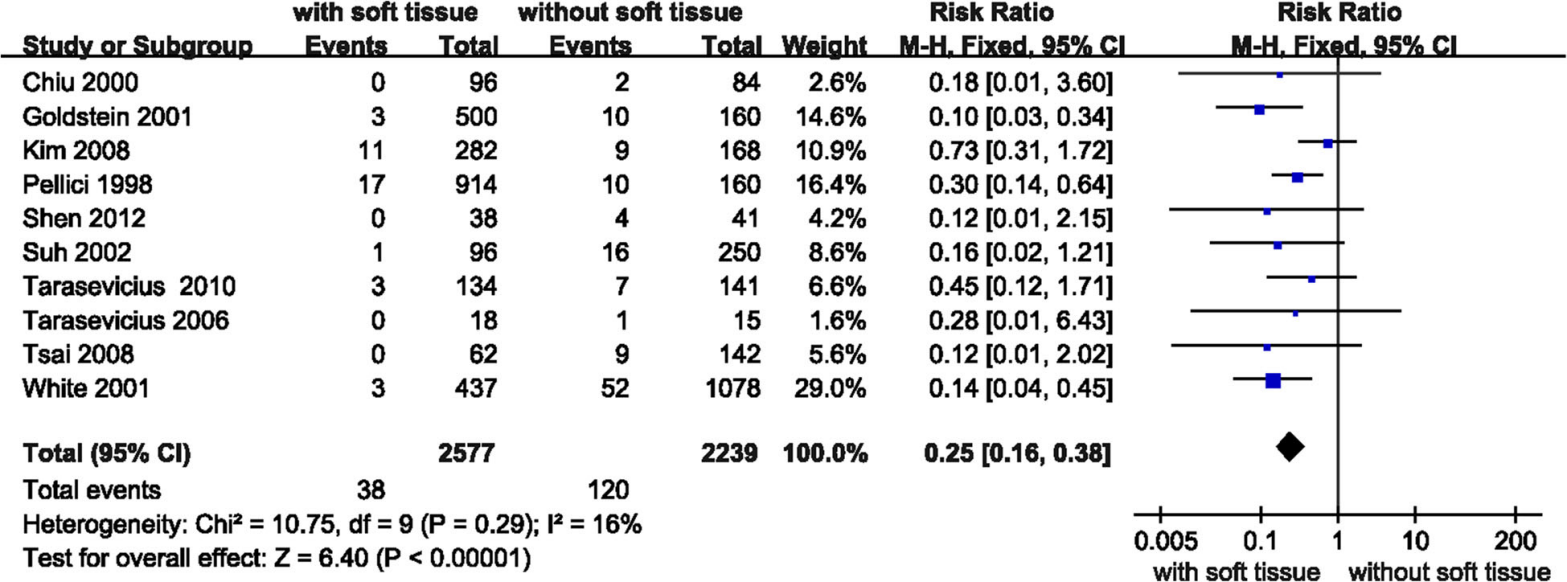
Forest plot of early dislocation rate between soft tissue repair group and no repair group

Eight studies were relevant to the analysis of the comparison of early dislocation rate between soft tissue repair group, the group in which both capsule and rotators were repaired, and no repair group (Fig. 4). Relative risk (RR) was used because it’s dichotomous outcome. Fixed effect model was chosen because there was no statistical evidence of heterogeneity (*P* = 0.29). Overall, the soft tissue repair group showed a significant lower early dislocation rate than no repair group. (RR = 0.25; 95% CI: 0.16–0.38; *P* = 0.0005; I^2^ = 16%)

Meta regression was performed to assess the effect of confounding factors, including ABA and ANA, in the occurrence of dislocation, No significant difference could be attributed to ABA (*p* = 0.25) or ANA (*p* = 0.16).

### Result 2: Comparison of early dislocation rate between soft tissue repair group and no repair group (RCT studies only)

**Fig. 5:**
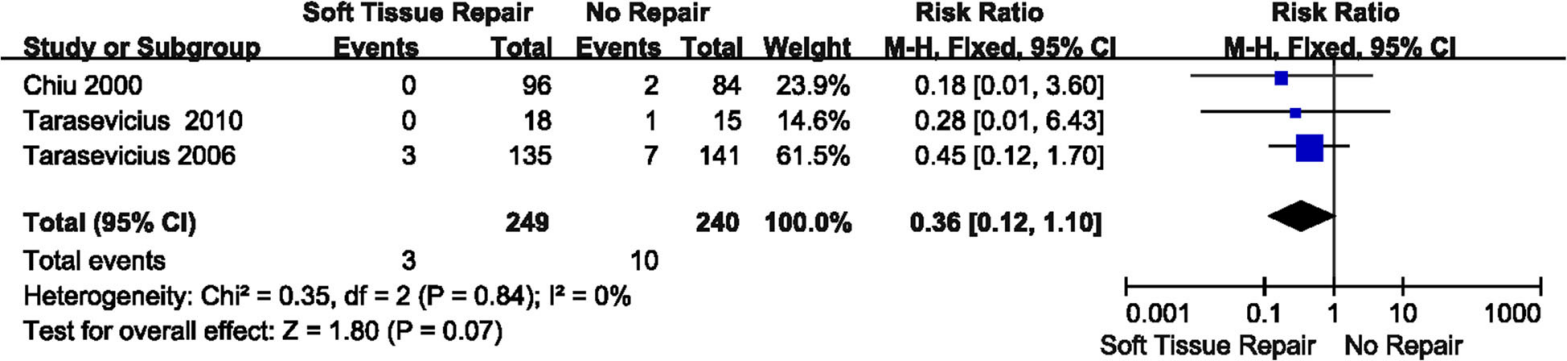
Forest plot of early dislocation rate between soft tissue repair group and no repair group (RCT studies only)

Three studies were relevant to the analysis of comparing early dislocation rate between soft tissue repair group and no repair group in only RCT studies (Fig. 5). Relative risk (RR) was used because it was dichotomous outcome. Fixed effect model was chosen because there was no statistical evidence of heterogeneity (*P* = 0.84 > 0.1). Overall, no significance was observed between the soft tissue repair group and no repair group in early dislocation rate in RCT studies only. (RR = 0.36; 95% CI: 0.12–1.10; *P* = 0.07; I^2^ = 0%)

### Result 3: Comparison of early dislocation rate between capsule repair group and no capsule repair group

**Fig. 6:**
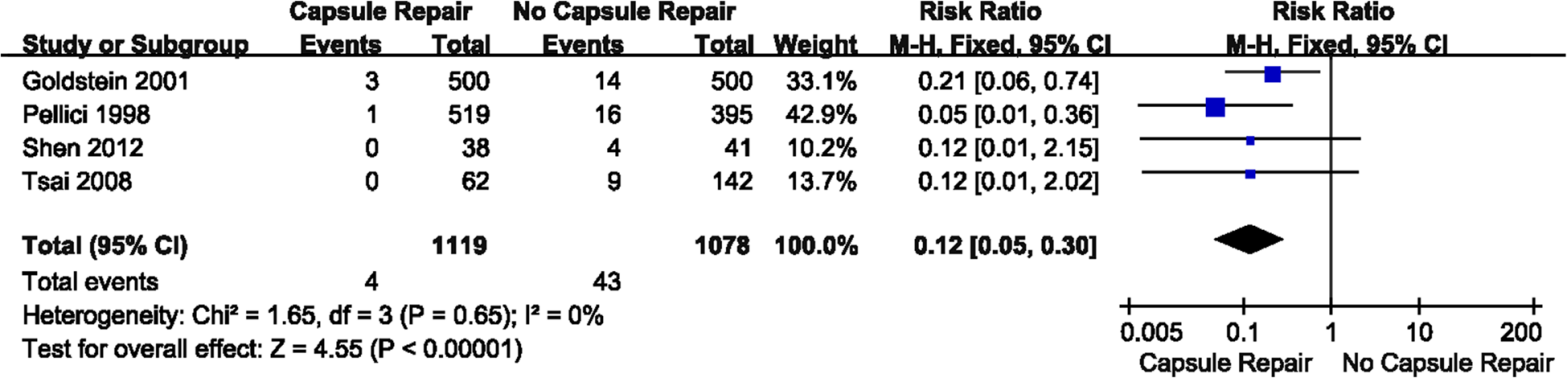
Forest plot of early dislocation rate between capsule repair group and no capsule repair group

Four studies were relevant to the analysis of comparison of early dislocation rate between capsule repair group and no capsule repair group (Fig. 6). Relative risk (RR) was used because of its dichotomous outcome. Fixed effect model was chosen because there was no statistical evidence of heterogeneity (*P* = 0.65 > 0.1). Overall, the capsule repair group showed a significant lower early dislocation rate than no capsule repair group. (RR = 0.12; 95% CI: 0.05–0.30; *P* < 0.00001; I^2^ = 0%).

### Result 4: Comparison of early dislocation rate between rotators repair group and no rotators repair group

**Fig. 7:**
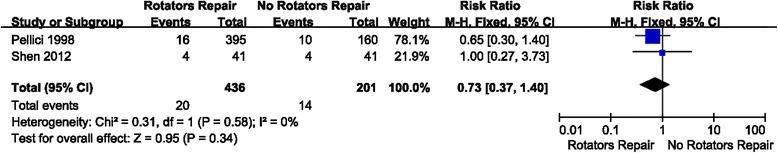
Forest plot of early dislocation rate between rotators repair group and no rotators repair group

Two studies were relevant to the analysis of comparison of early dislocation rate between rotators repair group and no rotators repair group (Fig. 7). Relative risk (RR) was used because it’s dichotomous outcome. Fixed effect model was chosen because there was no statistical evidence of heterogeneity (*P* = 0.58 > 0.1). Overall, no significance was observed between the rotators repair group and no rotators repair group in regards to the early dislocation rate. (RR = 0.73; 95% CI: 0.37–1.40; *P* < 0.34; I^2^ = 0%).

### Result 5: Comparison of HSS score between soft tissue repair group and no repair group

**Fig. 8:**
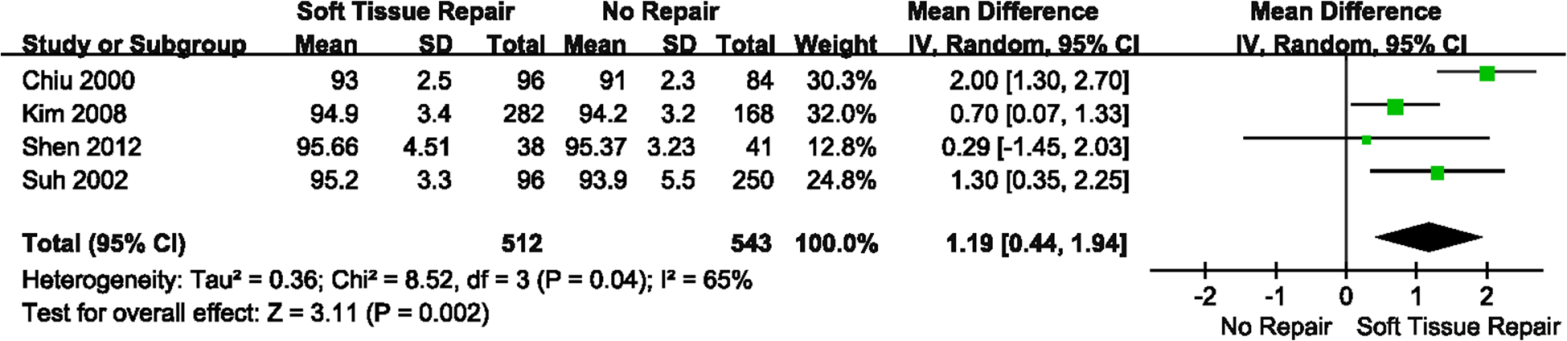
Forest plot of HSS score between soft tissue repair group and no repair group

Four studies were relevant to the analysis of comparison of HSS score between soft tissue repair group and no repair group (Fig. 8). Mean Difference (MD) was used because it is continuous outcome. Radom effect model was chosen because there was no statistical evidence of heterogeneity (*P* = 0.04 < 0.1). Overall, a significant higher HSS score was observed in soft tissue repair group than no repair group. (MD = 1.19; 95% CI: 0.44–1.94; *P* = 0.002; I^2^ = 65%)

### Results 6: Comparison of other complications (except early dislocaton) between soft tissue repair group and no repair group

In all included studies, 4 greater trochanter fracture, 2 sciatic nerve palsies and 1 infection were reported in soft tissue repair group while no cases were observed in the no repair group.

## Discussion

With the progress of surgical technique in recent years, THA has been an important and common treatment for aseptic necrosis of head of femur, rheumatoid hip arthritis, hip osteoarthritis, fracture of neck of femur, developmental dysplasia of the hip and some hip tumor. Meanwhile, increasing number of THA cases have been problematic all over the world, and more cases with sorts of complications have been reported. Early dislocation is one of the most serious complication after THA, an occurrence second to septic prosthesis loosing [14]. Reducing the incidence of hip dislocation is a significant issue to an orthopedist.

Generally, hip dislocation after THA is influenced by multiple factors, which could be mainly divided into three aspects [1]: (1) Patient related factors, (2) Postoperative management and (3) Surgery related factors. Patient related factors include age, gender, original disease and surgery history of hip. It’s reported that dislocation in aged patients over 80 was 2–3 times than usual [15, 16]. It’s also reported the incidence of dislocation rate in female patients was about twice than the rate compared to male patients [17]. Dislocation rate also increased when patients had a history of hip surgery or nervous system related diseases such as Parkinson’s disease [18]. Postoperative management was also important for patients after THA. Patients should be informed to avoid doing high-risk postures.

Surgery related factors included surgery approach, experience, prosthesis position, offset, and prosthesis design. Different surgery approaches could result in different dislocation rates [19]. However only posterior approach was analyzed in this study. The experience of surgeon was directly related to the dislocation rate. The reason that dislocation rate of decreased from 0.8 to 0.4% in ten years was because the surgeons become more experienced with the number of THA cases they conducted [7]. The position of prosthesis is the most important factor for the stability of the joint after surgery. The optimal abduction angle is 40° ± 10° and anteversion angle is 15° ± 10°. Lewinnek reported the dislocation increased fourfold when the parameters were out of that range [20]. In our study, all included literatures didn’t report a significant difference in ABA and ANA among groups, which suggested good control of confounding factors. In addition, a meta regression was performed to analyze the heterogeneity in dislocation among included studies that could be attributed to ABA and ANA, and no statistical significance was found. Offset is also a key factor of hip stability because it is the force arm of hip abductor which is a key muscle that keeps the hip stable. Prosthesis design also matters in the incidence of hip dislocation, especially the diameter of femur head. It was reported that the dislocation rate decrease significantly when the diameter of femur head was over 28 mm [21]. The head-to-neck ratio of prosthesis is important too, it’s reported that components with higher ratios impinge less [1].

Soft tissue repair is also a surgical related factors that affected the hip dislocation rate after THA. However, its real efficacy is still controversial. This meta-analysis included 10 clinical trials including 4816 hips treated with posterior approach in primary THA to assess its efficacy. Parimarily, according to all included data, the results demonstrated a significant lower early dislocation rate and higher HSS score in soft tissue repair group compared to no repair group. It is consistent with previous meta-analysis, which means soft tissue repair may be an effective method to decrease hip dislocation. However, as 7 of 10 included studies were retrospective studies, we did a subgroup analysis from 3 RCTs. It was interesting that there was no statistical significance observed in dislocation rate at this time. That means the effect of soft tissue repair to avoid hip dislocation is still not conclusive although it worked in many published retrospective studies. On the other hand, Kim et al. [9] and Suh et al. [10] reported soft tissue repair would increase operation time, which is not surprising as the results in additional steps to suture soft tissue including capsule and rotators. It is necessary to mention that longer operation time may result in higher risk of complications from anesthesia [22]. We also noticed that there were 4 greater trochanter fractures, 2 sciatic nerve palsies and 1 infection reported in soft tissue repair group while no cases were observed in the no repair group in all included studies. Asymptomatic avulsion fracture of the greater trochanter could be induced by decreasing mechanical strength during reattachment of capsule flap and increasing tension of soft tissue when patients exceed the available ROM postoperatively, and a modification of tension reducing and strength enhancing technique was raised by the original author to avoid this peculiar complication. Besides, sciatic nerve damage could be directly related to the tendon reconstruction, which is another issue should be taken into consideration. Under the circumstances, safety should be a crucial issue for soft tissue repair, but related complications could be avoided via cautious operation and improved operation technique, and shouldn’t be the obstacle for this technique.

Besides, two more subgroup analysis were made to analyze the effect of capsule repair only or rotators repair only. We found that the effect of capsule repair to prevent early dislocation was significant while rotators repair was not, which means capsule repair played a greater role in forming a mechanical barrier and decreasing dislocation rate than rotators repair. Also from our experience, method of rotators repair was more complicated than capsule repair which could spend more time and have higher risk to damage sciatic nerve, but it still needs to be confirmed in the future research. Capsule repair seems a safe trial and effective method to avoid hip dislocation in some extent, but it is important to figure out the function of capsule especially the mechanical property when deciding whether repair capsule, because capsule function may get worse because of aging. So it is an interesting issue to determine the relationship between aging and capsule function in the future.

This is an updated meta-analysis for this issue, but it is the first one to compare the effect of soft tissue repair, capsule repair and rotators repair separately, and the first time safety issue has been mentioned in soft tissue repair method. One limitation of this study is that in the included studies there were not enough RCTs, which may affect the quality of our meta-analysis. Although we have included all related studies thus far and tried to collect more data to make this meta-analysis and assess its effect, more research is needed to confirm the results and conclusions. Another limitation is the lack of detailed information reported about acetabulum prosthesis management and size of femur head in included studies, and many different kinds of prosthesis were used in each study which may increase the heterogeneity.

## Conclusions

Overall, according to results above, we conclude that although soft tissue repair could build mechanical barrier to prevent dislocating after THA, and it seemed to work in several studies and also resulted in better HSS score. However, a stricter meta-analysis showed the efficacy of soft tissue repair is still not clear on decreasing dislocation rate which more RCTs are needed to confirm. It is also not negligible that more complications such as greater trochanter fractures, sciatic nerve palsies and infections were found in soft tissue repair, meanwhile more steps could extend the surgery. So it is recommended to think twice to decide whether to perform soft tissue repair until more high quality studies are published to draw conclusive results. Additionally, it’s interesting that capsule repair only showed a better result than rotators repair only. Given the method to repair capsule is simpler, capsule repair only may be a safe and effective method to use in THA, but the issue of aging should be considered because it may affect the function of capsule.

## Abbreviations

ABA: Abduction angle
ANA: Anteversion angle
CI: Confidence intervals
HSS: Hospital of special surgery
MD: Mean differences
RCT: Randomized control trial
RR: Relative risk
THA: Total hip arthroplasty
